# Efficacy and safety of peppermint oil for the treatment in Japanese patients with irritable bowel syndrome: a prospective, open-label, and single-arm study

**DOI:** 10.1186/s13030-024-00302-y

**Published:** 2024-02-08

**Authors:** Kei Matsueda, Shin Fukudo, Masayuki Ogishima, Yuki Naito, Soichiro Nakamura

**Affiliations:** 1 Sakura Life Clinic, Tokyo, Japan; 2 Department of Behavioral Medicine, Tohoku University Graduate School of Medicine, Sendai, Japan; 3 Zeria Pharmaceutical Co., Ltd., 10-11 Nihonbashikobuna-Cho, Chuo-Ku, Tokyo, 103-8351 Japan

**Keywords:** Clinical trial, Herbal medicine, Irritable bowel syndrome, Japanese patients, Peppermint oil, Rome III criteria, ZO-Y60

## Abstract

**Background:**

In Europe, an herbal medicine containing peppermint oil is widely used in patients with irritable bowel syndrome (IBS). In Japan, however, no clinical evidence for peppermint oil in IBS has been established, and it has not been approved as a drug for IBS. Accordingly, we conducted a clinical study to confirm the efficacy and safety of peppermint oil (ZO-Y60) in Japanese patients with IBS.

**Methods:**

The study was a multi-center, open-label, single-arm, phase 3 trial in Japanese outpatients with IBS aged 17–60 years and diagnosed according to the Rome III criteria. The subjects were treated with an oral capsule of ZO-Y60 three times a day before meals, for four weeks. The efficacy of ZO-Y60 was evaluated using the patient’s global assessment (PtGA), IBS symptom severity score, stool frequency score, stool form score, and physician’s global assessment (PGA). The safety of ZO-Y60 was also assessed.

**Results:**

Sixty-nine subjects were treated with ZO-Y60. During the four-week administration of ZO-Y60, the improvement rate of the PtGA was 71.6% (48/67) in week 2 and 85.1% (57/67) in week 4. It was also suggested that ZO-Y60 is effective against any type of IBS (IBS with constipation, IBS with diarrhea, and mixed/unsubtyped IBS). The improvement rate of the PGA was 73.1% (49/67) in week 2 and 85.1% (57/67) in week 4, also confirming the efficacy of ZO-Y60. Adverse events were observed in 14 subjects (20.3%), however, none of these adverse events were categorized as serious.

**Conclusion:**

The efficacy of treatment was confirmed, subjective symptoms were improved, as was observed in previous clinical studies of ZO-Y60 conducted outside of Japan. All adverse reactions were previously known and were non-serious. These findings suggest that peppermint oil may be effective in the Japanese population and that it has an acceptable safety profile.

**Trial registration:**

JAPIC Clinical Trials Information number: JapicCTI-121727 https://jrct.niph.go.jp/en-latest-detail/jRCT1080221685. Registration date: 2012–01-10.

## Introduction

Irritable bowel syndrome (IBS) is recognized as a representative disorder of gut-brain interaction which is characterized by persistent gastrointestinal symptoms without major organic diseases detected by routine clinical examinations [1]. According to the Rome IV criteria, IBS is defined as recurrent abdominal pain at least once day or more per one week in each of the preceding three months and this pain meets two or more of the following: 1) associated with defecation, 2) onset associated with a change in frequency of bowel movement, and/or 3) onset associated with a change in form (appearance) of stool [2]. Moreover, the IBS symptoms must have started at least six months or more before the diagnosis. IBS is divided into IBS with predominant constipation (IBS-C), IBS with predominant diarrhea (IBS-D), IBS with mixed bowel habits (IBS-M), and IBS unsubtyped (IBS-U). The global prevalence of IBS using the Rome III and Rome IV criteria is 10.1% and 4.1%, respectively, and in Japan it is 9.3% and 2.2%, respectively [3]. Thus, IBS is a common disease worldwide, and stress is known to both trigger and worsen IBS. As a result it is often regarded as a “modern disease” that is expected to increase in frequency in the future. IBS has a large impact on the quality of life of patients and medical costs in advanced countries [4]. The cause of IBS remains to be fully clarified. It is currently treated not by curative therapy but symptomatic therapy. Effective treatments for IBS needs to be developed.

The Japanese guidelines for diagnosis and treatment for IBS at the first step provide instructions on how to improve meal composition and lifestyle choices, based on the predominant symptom: diarrhea, constipation or abdominal pain [1]. A macromolecular polymer or gastrointestinal motility regulator should then be administered to control the water content of the stool. Moreover, according to the predominant symptom, the following medications may be administered; a *Lactobacillus* preparation for diarrhea, a laxative for constipation, and an anticholinergic drug for abdominal pain. When stress or a psychological condition such as depression or anxiety plays a large part, an antidepressant or non-benzodiazepine anxiolytic is administered as a second step. For patients who are refractory to the pharmacotherapy, psychotherapy is recommended as the third step. These steps are clinically relevant and practical for most patients with IBS.

The use of peppermint oil is a unique approach to IBS treatment. Regarding its mechanism of action, the major component l-menthol exerts its effect by inhibiting smooth muscle contraction by blocking Ca^2+^ channels [5]. Several randomized controlled trials have confirmed that peppermint oil is effective for the relief of the symptoms in IBS patients [6–11]. These findings were confirmed by systematic reviews and meta-analyses [12, 13]. Based on this evidence, the European, Canadian, and Japanese clinical practice guidelines recommend peppermint oil to alleviate IBS symptoms [1, 14, 15]. ZO-Y60 is an enteric-coated capsule, the active ingredient of which is peppermint oil extracted from the peppermint plant (*Mentha x piperita L*.). Outside Japan, ZO-Y60 has been tested and shown to improve IBS symptoms in several double-blind, placebo-controlled trials involving IBS patients [8–11]. However, there is currently no published data on the use of peppermint oil as therapy for patients with IBS in Japan.

A summary of approval criteria for herbal remedies as outlined in 2007 in the Application Guideline for Western Traditional Herbal Medicines as OTC Drugs [16] includes the following:

- OTC drug approval under the pharmaceutical regulation of certain Western countries.
- Efficacy and safety based on the scientific evaluation of well-designed clinical trials including some clinical literature.
- Efficacy and safety data already assessed in an acknowledged country that can be utilized in the approval process in Japan.
- Quality comparable to drug products used in clinical trials to show their efficacy and safety as indicated above.
- Safety in the Japanese population is required.

We judged that ZO-Y60 met the above guideline and planned a non-controlled open-label trial with the main objective of confirming its safety and to study its efficacy in a real-world population of Japanese IBS patients.

## Methods

### Subjects

The patients screened were those with IBS as defined by the Rome III criteria [17] because the trial was conducted before the Rome IV criteria were issued. We enrolled patients who had visited one of three medical institutions between January and June 2012. Major inclusion criteria were as follows: 1) outpatients aged between 15 and 64 years when consenting; 2) patients who could understand the contents of this clinical trial and who have personally given written consent to participate in the clinical trial; and 3) patients with abdominal pain or abdominal discomfort for two days or more during the seven-day screening period (the seven days just prior to the day of determination of final enrollment). Major exclusion criteria were as follows: 1) patients who exhibited persistent fever or arthralgia or hematochezia or had experienced unexpected weight loss of 3 kg or more within the preceding six months; 2) those who had a history of a malignant tumor or were receiving treatment for this condition; 3) those receiving treatment for ischemic colitis; 4) those receiving treatment for infectious colitis; 5) those with a history of inflammatory bowel disease or were receiving treatment for this condition; 6) those exhibiting organic disease (excluding any diverticulum of the large intestine or a polyp of 5 mm or smaller considered to have no impact on gastrointestinal transit) on total colonoscopy, performed on the day of determination of final enrollment; and 7) those adjudged to have hyperthyroidism or hypothyroidism based on laboratory values of thyroid stimulating hormone, free T3, and free T4 on the day of determination of tentative enrollment.

The target sample size was set at sixty as the number of cases for which safety evaluation was possible, referring to the major overseas clinical studies of this drug [8].

All participants provided written informed consent prior to study participation, and the study protocol was approved by the ethics committee at Fukui General Hospital (ZO-Y60 20111202). The trial was performed in accordance with the Declaration of Helsinki and subsequent revisions. The trial was registered at Japic Clinical Trials Information (JapicCTI-121727).

### Study design

**Fig. 1:**
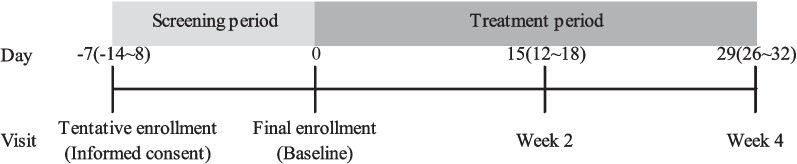
Study period

The study was designed as multi-center, non-controlled, open-label trial. The investigational drug, ZO-Y60, is an enteric-coated capsule containing 187 mg (0.2 mL) of peppermint oil per capsule. The drug was administered orally before meals, three times a day for a duration of four weeks. A one-week screening period was established between the day of determination of tentative enrollment (the day of consent) and that of determination of final enrollment. During this period, the investigational drug was not administered. The treatment period lasted four weeks from the day after the determination of final enrollment. The intervals in the clinical trial are shown in Fig. 1.

The investigator checked whether any pretreatment drug, pretreatment therapy, or health food was used to treat IBS within the 28 days before the determination of tentative enrollment. If any prohibited concomitant drug or health food was used, it was washed out after the determination of tentative enrollment. Before the determination of final enrollment, the investigator confirmed that no treatment had been conducted for 10 days or more just prior to the day of determination of final enrollment. If the study treatment was discontinued, observations and examinations specified at discontinuation of the treatment period were conducted whenever possible between the day of discontinuation (designated as Day 0) and Day 7.

On determining tentative enrollment, the investigator interviewed the subject to determine the subtype of their IBS based on their stool consistency using the Bristol Stool Form Scale (BSFS); 1: lumpy, 2: aggregated lumpy, 3: hard with fissures, 4: sausage-like, 5: soft blobs, 6: mushy, 7: watery) during the past three months. Determination of the subtype was based on the Rome III criteria [17]. In brief, IBS-C; a patient with BSFS type 1 or 2 ≧25% and type 6 or 7 < 25%, IBS-D; a patient with BSFS type 6 or 7 ≧25% and type 1 or 2 < 25%, IBS-M; a patient with BSFS type 6 or 7 ≧25% and type 1 or 2 ≧25%, and IBS-U; a patient who satisfies neither IBS-C, IBS-D, nor IBS-M [17].

### Concomitant drugs, therapies, and other prohibited agents

During the period of the clinical trial, the combination of ZO-Y60 and any of the drugs or therapies listed below was prohibited because these agents were considered to have an impact on the assessment of the efficacy or safety of ZO-Y60: any therapeutic agent for IBS, prokinetic agent, anticholinergic agent, cholinergic agent, spasmolytic, psychotropic agent, sedative-hypnotic, anti-anxiety agent, antidiarrheal drug, drug for controlling intestinal function, cathartic, enema, gastric secretion inhibitor, antacid, antipyretic analgesic and anti-inflammatory agent, narcotic drug/narcotic antitussive, macrolide antibiotic preparation, Chinese herbal medicine, health food containing peppermint oil as the active ingredient, psychotherapy, and dietetic therapy. However, an antipyretic analgesic and anti-inflammatory agent was permitted to be used to treat a comorbidity or adverse events for up to four days in total, but only during the treatment period.

### Efficacy assessment

Because the Pharmaceuticals and Medical Devices Agency of Japan approved the setting of responder rates for global assessment as the primary endpoint in IBS trial in 2002 [18, 19], the patient’s global assessment (PtGA) was also assessed as one of the efficacy endpoints in this trial. Other efficacy measures were the improvement rate of the physician’s global assessment (PGA) and change in IBS symptom severity score. The subjects rated the degree of the PtGA on a four-point scale, comparing “the seven days immediately prior to the day of determination of final enrollment” (the screening period) and “the seven days immediately prior to each investigation”, recording this in a questionnaire. The four-point scale for the degree of the PtGA was as follows: 2, the IBS symptoms have been greatly improved from the screening period; 1, the IBS symptoms have been improved from the screening period; 0, the IBS symptoms have not changed from the screening period; and − 1, the IBS symptoms have worsened from the screening period. On the visit days for week 2 and week 4 of the treatment period (or at discontinuation of the treatment period), the subjects with the degree of the PtGA “2” or “1” were designated as “subjects with improvement”. The proportion of such subjects in the analysis set was designated “the improvement rate of the PtGA”. Rating of PGA was conducted as was the rating of the PtGA.

The endpoints were then analyzed. Rating of IBS symptom severity by the subjects was as follows. In a symptom and medication diary, the subjects recorded the severity of each of their IBS symptoms every day before bedtime (except the day of determination of final enrollment), from the day of determination of tentative enrollment to the day before the visit during week 4 of the treatment period (or at discontinuation of the treatment period). Symptoms included abdominal pain or abdominal discomfort, abdominal distension, borborygmi, flatulence, sensation of incomplete defecation, defecation urgency, straining at defecation, belching, nausea, and heartburn. The subjects assessed the worst severity of each symptom every day on a four-point scale of IBS symptom severity, as follows: 0, no (no symptoms); 1, mild (symptom not affecting daily life); 2, moderate (symptom affecting daily life but causing minimal change); and 3, severe (symptom having a substantial effect on daily activities). The mean severity of each IBS symptom during the 7 days before the visit days for determination of final enrollment, week 2, and week 4 of the treatment period (or at discontinuation of the treatment period) was used as “IBS symptom severity score”, and the difference in the IBS symptom severity score obtained at the visit for determination of final enrollment and that for week 2 or week 4 of the treatment period (or at discontinuation of the treatment period) was calculated and used as “change in IBS symptom severity score” at the time of each investigation.

Evaluation of stool frequency and form by subjects was as follows. In a diary of symptoms and dosages, the subjects recorded daily stool frequency and the most notable (most troublesome) stool form every day before retiring (except the day of determination of final enrollment) from the day of determination of tentative enrollment to the day before the visit during week 4 of the treatment period (or at discontinuation of the treatment period). The stool form was rated based on the BSFS. The mean daily stool frequency during the 7 days before the visit days for determination of final enrollment, week 2, and week 4 of the treatment period (or at discontinuation of the treatment period) was used as the “stool frequency score”. The difference in the stool frequency score obtained at the visit for determination of final enrollment and that for week 2 or week 4 of the treatment period (or at discontinuation of the treatment period) was calculated and used as the “change in stool frequency score” at the time of each investigation. The numeric stool form ratings of the BSFS [17] for the 7 days before the visit days for determination of final enrollment, week 2, and week 4 of the treatment period (or at discontinuation of the treatment period) were summed, and their mean was used as the “stool form score”. The difference in the stool form score obtained at the visit for determination of final enrollment and that for week 2 or week 4 of the treatment period (or at discontinuation of the treatment period) was calculated and used as the “change in stool form score” at the time of each investigation.

If treatment was discontinued, any endpoint assessment that occurred involved comparison between the screening period and the seven days immediately prior to discontinuation. If the duration from the day when the investigational drug was started to the day of discontinuation was less than seven days, this duration was used for assessment.

### Safety assessment

To assess for adverse events, the investigator performed clinical laboratory tests and physical examinations at the time of investigations. Clinical laboratory tests included hematology tests (white blood cell count, red blood cell count, hemoglobin, hematocrit, and platelet count), biochemical tests (total protein, albumin, AST, ALT, LDH, ALP, γ-GTP, total bilirubin, direct bilirubin, creatinine, uric acid, urea nitrogen, total cholesterol, Na, K, and Cl), and general urinalyses (protein qualitative, sugar qualitative, and urobilinogen qualitative). Physical examinations included body temperature, blood pressure, and pulse rate. Adverse events were defined as all unfavorable or unintentional signs, symptoms, or illness occurring after administration of the investigational drug. Adverse events judged to “have a causal relationship to the investigational drug” were designated as adverse reactions. The incidence of each adverse event/reaction was evaluated.

### Statistical analyses

This study is not a confirmatory trial, thus, no verifiable hypothesis was established. The efficacy analysis set was composed of IBS patients who had efficacy data after the study treatment. The safety analysis set was composed of subjects who had safety data after the study treatment. When a test was performed, the significance level (two-sided) was set at 0.05, and statistical significance was confirmed with “*p* < 0.05”. When a confidence interval (CI) was determined, the lower and upper limits (two-sided) were set at 0.95. SAS Release 9.2 (SAS Institute Inc., Cary, NC, USA) was used for all statistical calculations. MedDRA/J Ver. 15.0 was used to code adverse event names before use in the counting and analysis.

## Results

### Participant flow

**Fig. 2:**
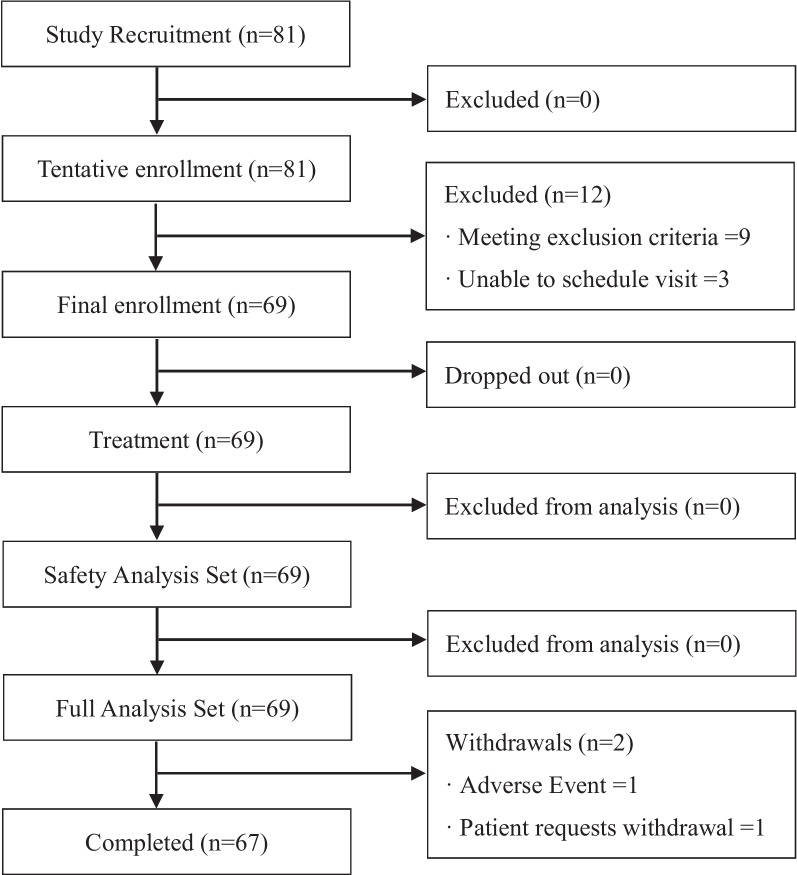
The flow diagram of the study

Eighty-one patients consented, and all were tentatively enrolled. Of these patients, 69 were finally enrolled: 12 did not qualify for the final enrollment, three failed to schedule a visit and nine met the exclusion criteria. Of the nine patients meeting the exclusion criteria, six exhibited organic disease at colonoscopy, one had suspected hyperthyroidism, one had suspected chronic thyroiditis, and the remaining patient withdrew, complaining of pain on insertion of the endoscope. All of the 69 enrolled patients were administered ZO-Y60, with 67 completing the study and two discontinuing, one exhibiting eczema as an adverse event and considered unable to continue participation in the clinical trial and the other for personal reasons (Fig. 2).

None of the subjects treated had serious concerns leading to withdrawal from any analysis set that was part of the clinical trial protocol or were excluded due to non-compliance. Thus, 69 subjects constituted the efficacy analysis (full analysis set) and safety analysis sets. The two subjects mentioned above had administration stopped on the sixth and seventh days after the start of investigational drug administration. Concerning the efficacy analysis, therefore, the data for these two subjects were excluded from the analysis at week 2 and week 4.

The summary statistics for baseline demographic and clinical characteristics are shown in Table 1. In total, there were 37 male and 32 female subjects. The subject age range was between 17 and 60 years. Mean age (standard deviation) was 37.8 years (11.8). Most of the subjects were in the 20–29 and 40–49 age groups and weighed not less than 50 kg and not more than 70 kg. IBS duration was as follows: not less than 6 months and less than 1 year, *n* = 4; not less than 1 year and less than 3 years, *n* = 13; not less than 3 years and less than 10 years, *n* = 22; and not less than 10 years, *n* = 30. IBS subtypes were as follows: IBS-C, *n* = 18; IBS-D, *n* = 35; IBS-M, *n* = 16; and unsubtyped IBS, *n* = 0. Two subjects had used over-the-counter pretreatment drugs. Before final enrollment, 13 subjects were adjudged to “have” organic disease as follows: 5 mm or smaller polyp only, *n* = 7; diverticulum of the large intestine only, *n* = 4; and both, *n* = 2.

**Table 1:** Baseline demographic and clinical characteristics (FAS)

Items		*N* = 69	(%)
Sex	Male	37	53.6
	Female	32	46.4
Age (years)	Mean	37.8	
	SD	11.8	
	Maximum	60	
	Median	39.0	
	Minimum	17	
	< 20	2	2.9
	20–29	20	29.0
	30–39	14	20.3
	40–49	19	27.5
	50–59	13	18.8
	≥ 60	1	1.4
Weight (kg)	Mean	61.29	
	SD	11.65	
	Maximum	87.0	
	Median	60.00	
	Minimum	39.0	
	< 50	13	18.8
	≥ 50– < 60	20	29.0
	≥ 60– < 70	21	30.4
	≥ 70– < 80	10	14.5
	≥ 80	5	7.2
Duration of illness	≥ 6 months– < 1 year	4	5.8
	≥ 1 year– < 3 years	13	18.8
	≥ 3 years- < 10 years	22	31.9
	≥ 10 years	30	43.5
IBS subtypes	IBS-C	18	26.1
	IBS-D	35	50.7
	IBS-M	16	23.2
	Unsubtyped	0	0.0
Pretreatment drugs	No	67	97.1
	Yes	2	2.9
Pretreatment therapy	No	69	100.0
	Yes	0	0.0
Intake of health food	No	69	100.0
	Yes	0	0.0
Organic disease^a^	No	56	81.2
	Yes	13	18.8
Medical history	No	51	73.9
	Yes	18	26.1
Concomitant medicine	No	63	91.3
	Yes	6	8.7
Concomitant therapy	No	69	100.0
	Yes	0	0.0

*SD* standard deviation

^a^Diverticulum of the large intestine or a 5 mm or smaller polyp

### Efficacy outcomes

#### Improvement rate of the patient’s global assessment (PtGA)

The degree of the PtGA: the frequency distribution, the number of subjects with improvement, the improvement rate, and its 95% CI are shown in Table 2. In week 2 of the treatment period, the number of subjects with improvement was 48, and the improvement rate (95% CI) was 71.6% (59.4, 81.9). In week 4 of the treatment period, the number of subjects with improvement was 57, and the improvement rate (95% CI) was 85.1% (74.3, 92.6). In week 4 of the treatment period, two subjects had a PtGA score of − 1, indicating that their IBS symptoms had worsened since the screening period; one of the two subjects reported an increased incidence of abdominal pain, defecation urgency, and loose stool, and the other subject reported loose stools persisting for several days. No symptom was severe enough to require treatment, and none were considered an adverse event.

**Table 2:** Improvement rate of the patient’s global assessment (PtGA) (FAS)

Visit	Degree of the patient’s global assessment				N	Improved cases^a^	%^b^(95% CI)
	-1	0	1	2			
Week 2	0	19	39	9	67	48	71.6 (59.4, 81.9)
Week 4	2	8	42	15	67	57	85.1 (74.3, 92.6)

^a^The total number at “2” or “1” degree of the patient’s global assessment

^b^The percentage of improved cases

#### Improvement rate of the physician’s global assessment (PGA)

The degree of the PGA: the frequency distribution, the number of subjects with improvement, the improvement rate, and its 95% CI are shown in Table 3. In week 2 of the treatment period, the number of subjects with improvement was 49, and the improvement rate (95% CI) was 73.1% (61.0, 83.2). In week 4 of the treatment period, the number of subjects with improvement was 57, and the improvement rate (95% CI) was 85.1% (74.3, 92.6). These results are similar to those of the PtGA.

**Table 3:** Improvement rate of the physician’s global assessment (PGA) (FAS)

Visit	Degree of the physician’s global assessment				N	Improved cases^a^	%^b^(95% CI)
	-1	0	1	2			
Week 2	0	18	40	9	67	49	73.1 (61.0, 83.2)
Week 4	2	8	39	18	67	57	85.1 (74.3, 92.6)

^a^The total number at “2” or “1” degree of the physician’s global assessment

^b^The percentage of improved cases

#### Change in IBS symptom severity score

The summary statistics for the change in IBS symptom severity score, 95% CI of the mean, and the results of a paired t-test are shown in Table 4. For administration week 2, the severity scores for the following seven symptoms were significantly lower than during the screening period: abdominal pain or abdominal discomfort, abdominal distension, borborygmi, flatulence, sensation of incomplete defecation, defecation urgency, and straining at stool. In week 4, the severity score for the above-mentioned seven symptoms as well as heartburn was significantly lowered. Concerning belching and nausea, the severity score did not change.

**Table 4:** Change in IBS symptom severity score (FAS)

IBS symptom	Visit	N^a^	Change in IBS symptom severity score					95% CI of mean	Paired t-tests
			Mean	SD	Minimum	Median	Maximum		
Abdominal pain or abdominal discomfort	Week 2	67	-0.53	0.53	-1.9	-0.60	0.9	-0.66, -0.41	*p* < 0.001
	Week 4	67	-0.57	0.70	-2.6	-0.60	1.4	-0.73, -0.40	*p* < 0.001
Abdominal distension	Week 2	62	-0.50	0.62	-2.3	-0.40	0.5	-0.65, -0.34	*p* < 0.001
	Week 4	62	-0.55	0.79	-2.3	-0.50	1.3	-0.74, -0.35	*p* < 0.001
Borborygmi	Week 2	58	-0.17	0.47	-1.9	-0.10	0.7	-0.29, -0.05	*p* = 0.007
	Week 4	58	-0.24	0.58	-1.9	-0.20	1.7	-0.39, -0.09	*p* = 0.002
Flatulence	Week 2	55	-0.27	0.57	-1.9	-0.10	0.9	-0.42, -0.12	*p* < 0.001
	Week 4	55	-0.39	0.76	-2.4	-0.30	1.7	-0.60, -0.19	*p* < 0.001
Sensation of incomplete defecations	Week 2	65	-0.26	0.58	-2.3	-0.20	1.0	-0.39, -0.12	*p* < 0.001
	Week 4	65	-0.39	0.60	-2.3	-0.30	0.6	-0.53, -0.24	*p* < 0.001
Defecation urgency	Week 2	58	-0.21	0.45	-1.7	-0.10	1.0	-0.32, -0.10	*p* < 0.001
	Week 4	58	-0.27	0.53	-2.0	-0.10	1.0	-0.40, -0.13	*p* < 0.001
Straining at stool	Week 2	59	-0.24	0.57	-1.9	-0.30	1.0	-0.38, -0.09	*p* = 0.002
	Week 4	59	-0.43	0.58	-2.3	-0.40	0.6	-0.57, -0.28	*p* < 0.001
Belching	Week 2	36	-0.03	0.49	-1.3	0.00	1.0	-0.19, 0.13	*p* = 0.737
	Week 4	36	-0.19	0.59	-1.8	-0.05	1.4	-0.38, 0.01	*p* = 0.065
Nausea	Week 2	26	-0.12	0.50	-1.8	-0.05	0.9	-0.31, 0.08	*p* = 0.231
	Week 4	26	-0.21	0.65	-2.4	-0.10	1.4	-0.47, 0.05	*p* = 0.109
Heartburn	Week 2	38	-0.13	0.55	-1.8	-0.10	1.1	-0.31, 0.05	*p* = 0.151
	Week 4	38	-0.23	0.65	-2.3	-0.10	1.3	-0.44, -0.02	*p* = 0.033

*SD* standard deviation

^a^For each symptom, subjects who reported that “the symptom did not develop” during the screening period or at the time of any investigation were excluded from the analysis set for calculation

#### IBS subtype rating

The degree of the PtGA in each visit for each IBS subtype: the frequency distribution, the number of subjects with improvement, the improvement rate, and its 95% CI are shown in Table 5. Regarding PtGA for each IBS subtype, the improvement rate was high regardless of the subtype: 66.7% to 81.3% for week 2 and 78.8% to 93.8% for week 4. The summary statistics of the stool frequency score and 95% CI of the mean for each IBS subtype are shown in Table 6. The summary statistics of the stool form score and 95% CI of the mean for each IBS subtype are shown in Table 7. The summary statistics of the change in stool frequency score, 95% CI of the mean, and the results of paired t-tests for each IBS subtype are shown in Table 8. The summary statistics of the change in stool form score, 95% CI of the mean, and the results of paired t-tests for each IBS subtype are shown in Table 9. The stool frequency score for IBS-C increased significantly from the screening period for both treatment periods: week 2 and week 4. The stool form score for IBS-D decreased significantly from the screening period to administration week 2.

**Table 5:** Improvement rate of the patient’s global assessment (PtGA) for each IBS subtype (FAS)

Visit	IBS subtypes	Degree of the patient’s global assessment				N	Improved cases^a^	%^b^(95% CI)
		-1	0	1	2			
Week 2	IBS-C	0	6	8	4	18	12	66.7 (41.0, 86.6)
	IBS-D	0	10	20	3	33	23	69.7 (51.3, 84.4)
	IBS-M	0	3	11	2	16	13	81.3 (54.4, 95.9)
Week 4	IBS-C	0	2	13	3	18	16	88.9 (65.3, 98.6)
	IBS-D	2	5	17	9	33	26	78.8 (61.1, 91.0)
	IBS-M	0	1	12	3	16	15	93.8 (69.8, 99.8)

^a^The total number of subjects with the degree of the patient’s global assessment of “2” or “1”

^b^The number of subjects with improvement as a percentage of all analysis subjects

**Table 6:** Stool frequency score for each IBS subtype (FAS)

IBS subtypes	Visit	N	Stool frequency score					95% CI of mean
			Mean	SD	Minimum	Median	Maximum	
IBS-C	Baseline	18	0.70	0.42	0.1	0.60	1.4	0.50, 0.90
	Week 2	18	0.91	0.40	0.3	0.90	2.0	0.71, 1.10
	Week 4	18	0.87	0.44	0.3	0.70	1.9	0.66, 1.09
IBS-D	Baseline	35	1.63	0.72	0.7	1.60	4.0	1.39, 1.88
	Week 2	33	1.64	0.72	0.4	1.60	4.1	1.39, 1.89
	Week 4	33	1.75	0.82	0.4	1.40	4.7	1.46, 2.04
IBS-M	Baseline	16	0.98	0.43	0.3	0.90	1.9	0.76, 1.21
	Week 2	16	1.11	0.37	0.6	1.00	1.9	0.92, 1.30
	Week 4	16	1.02	0.39	0.6	0.95	1.7	0.82, 1.22
Total	Baseline	69	1.24	0.72	0.1	1.10	4.0	1.07, 1.41
	Week 2	67	1.32	0.66	0.3	1.10	4.1	1.16, 1.47
	Week 4	67	1.34	0.76	0.3	1.30	4.7	1.16, 1.52

*SD* standard deviation

**Table 7:** Stool form score for each IBS subtype (FAS)

IBS subtypes	Visit	N	Stool form score					95% CI of mean
			Mean	SD	Minimum	Median	Maximum	
IBS-C	Baseline	18	3.23	1.11	1.2	3.05	5.0	2.68, 3.78
	Week 2	18	3.45	1.29	1.0	3.55	6.5	2.81, 4.09
	Week 4	18	3.32	1.06	1.0	3.55	5.3	2.79, 3.84
IBS-D	Baseline	35	4.83	0.63	2.4	4.90	6.0	4.62, 5.05
	Week 2	33	4.42	0.76	2.2	4.50	5.9	4.15, 4.68
	Week 4	33	4.66	0.68	3.4	4.70	5.8	4.43, 4.90
IBS-M	Baseline	16	4.06	1.37	2.0	4.00	6.7	3.33, 4.78
	Week 2	16	4.16	0.88	2.7	4.25	5.7	3.70, 4.63
	Week 4	16	4.09	0.95	2.0	4.00	5.8	3.59, 4.59
Total	Baseline	69	4.23	1.18	1.2	4.50	6.7	3.96, 4.51
	Week 2	67	4.10	1.03	1.0	4.30	6.5	3.85, 4.34
	Week 4	67	4.17	1.02	1.0	4.20	5.8	3.92, 4.41

*SD* standard deviation

**Table 8:** Change in stool frequency score for each IBS subtype score (FAS)

IBS subtypes	Visit	N	Change in stool frequency score					95% CI of mean	Paired t-tests
			Mean	SD	Minimum	Median	Maximum		
IBS-C	Week 2	18	0.21	0.34	-0.4	0.20	1.0	0.04, 0.37	*p* = 0.020
	Week 4	18	0.17	0.30	-0.5	0.20	0.7	0.03, 0.32	*p* = 0.026
IBS-D	Week 2	33	-0.02	0.42	-1.1	0.00	0.7	-0.16, 0.13	*p* = 0.835
	Week 4	33	0.09	0.57	-0.9	0.10	1.5	-0.10, 0.29	*p* = 0.350
IBS-M	Week 2	16	0.13	0.43	-0.9	0.25	0.9	-0.10, 0.36	*p* = 0.245
	Week 4	16	0.04	0.42	-1.0	0.05	0.6	-0.18, 0.26	*p* = 0.725
Total	Week 2	67	0.08	0.41	-1.1	0.10	1.0	-0.02, 0.17	*p* = 0.117
	Week 4	67	0.10	0.47	-1.0	0.10	1.5	-0.01, 0.21	*p* = 0.083

Amount of change in stool frequency score = “frequency at 2, 4 weeks after treatment (or at the end of treatment)”– “frequency at baseline”

*SD* standard deviation

**Table 9:** Change in stool form score for each IBS subtype score (FAS)

IBS subtypes	Visit	N	Change in stool form score					95% CI of mean	Paired t-tests
			Mean	SD	Minimum	Median	Maximum		
IBS-C	Week 2	18	0.22	1.45	-1.7	0.05	4.5	-0.49, 0.94	*p* = 0.524
	Week 4	18	0.09	0.85	-1.1	0.00	2.3	-0.33, 0.51	*p* = 0.662
IBS-D	Week 2	33	-0.39	0.65	-2.2	-0.30	0.9	-0.61, -0.16	*p* = 0.001
	Week 4	33	-0.14	0.67	-1.7	-0.10	1.0	-0.38, 0.09	*p* = 0.233
IBS-M	Week 2	16	0.11	1.29	-1.6	0.05	2.7	-0.57, 0.79	*p* = 0.745
	Week 4	16	0.04	1.23	-2.6	0.00	1.9	-0.61, 0.69	*p* = 0.904
Total	Week 2	67	-0.11	1.10	-2.2	-0.30	4.5	-0.37, 0.16	*p* = 0.431
	Week 4	67	-0.04	0.87	-2.6	0.00	2.3	-0.25, 0.17	*p* = 0.727

Amount of change in stool form score = “stool form score at 2, 4 weeks after treatment (or at the end of treatment)”– “stool form score at baseline”

*SD* standard deviation

### Safety outcomes

In the safety analysis set consisting of 69 subjects, the mean compliance (SD) over the entire period was 96.2% (4.65). The number of subjects with adverse events, the number of adverse events, and the number of subjects with adverse events as a percentage of the number of subjects analyzed (the incidence) for each adverse event/reaction are shown in Table 10. Adverse events were observed in 14/69 subjects (20.3%). All of these adverse events were classified as mild or moderate in severity, and neither severe nor serious adverse events were observed. Adverse reactions were observed in 2/69 subjects (2.9%). One exhibited eczema, and the other exhibited two events: breath odor and hypersensitivity. The eczema was moderate and led to discontinuation of the trial. The breath odor and hypersensitivity were mild. These adverse reactions resolved during the study period.

**Table 10:** Adverse events and advers drug reaction (SAF)

	*N* = 69			
System Organ Class	AEs	(%)	ADRs	(%)
Preferred Term				
Overall	14	(20.3)	2	(2.9)
Infections and Infestations	5	(7.2)	0	(0.0)
Nasopharyngitis	4	(5.8)	0	(0.0)
Otitis media	1	(1.4)	0	(0.0)
Immune system disorders	1	(1.4)	1	(1.4)
Hypersensitivity	1	(1.4)	1	(1.4)
Respiratory, thoracic and mediastinal disorders	1	(1.4)	0	(0.0)
Upper respiratory tract inflammation	1	(1.4)	0	(0.0)
Gastrointestinal disorders	1	(1.4)	1	(1.4)
Breath odour	1	(1.4)	1	(1.4)
Hepatobiliary disorders	1	(1.4)	0	(0.0)
Hepatic function abnormal	1	(1.4)	0	(0.0)
Skin and subcutaneous tissue disorders	1	(1.4)	1	(1.4)
Eczema	1	(1.4)	1	(1.4)
Renal and urinary disorders	1	(1.4)	0	(0.0)
Hypertonic bladder	1	(1.4)	0	(0.0)
General disorders and administration site conditions	1	(1.4)	0	(0.0)
Pyrexia	1	(1.4)	0	(0.0)
Investigations	5	(7.2)	0	(0.0)
Gamma-glutamyltransferase increased	2	(2.9)	0	(0.0)
Blood pressure increased	1	(1.4)	0	(0.0)
Glucose urine present	1	(1.4)	0	(0.0)
Blood alkaline phosphatase increased	1	(1.4)	0	(0.0)

Event name: MedDRA/J Ver. 15.0

*AE* Adverse event, *ADR* Adverse drug reaction

If a subject exhibited the same event more than once, one was added to the number of subjects with this event.

#### Counting of overall events

If a subject exhibited multiple events, one was added to the number of subjects with events.

## Discussion

Several clinical studies of peppermint oil have been conducted in patients with IBS outside Japan and the results demonstrated its efficacy and safety [6–13]. No study results, to date, have been published in Japanese patients. Therefore, we conducted a non-controlled, open-label trial to confirm its efficacy and safety in Japanese patients. The outcomes obtained in this study seemed to be comparable to those of a pivotal clinical study of ZO-Y60 conducted outside Japan [8, 10]. In our study, however, no control arm was established, only a single arm was treated with the active drug. Thus, the outcomes need to be carefully interpreted.

In this clinical trial, the improvement rate of the PtGA was 71.6% for week 2 and 85.1% for week 4 of the treatment period. The improvement rate of the PGA was 73.1% for week 2 and 85.1% for week 4 of the treatment period, similar to the improvement rate of the PtGA. The improvement rate of the PtGA was also evaluated for each IBS subtype. Regardless of the subtype, the improvement rate was high: 78.8% to 93.8% for week 4. According to the monograph of the European Medicines Agency’s HMPC (Committee on Herbal Medicinal Products), peppermint oil usually improves symptoms after one to two weeks of use [20]. In this clinical study, a high improvement rate was obtained at week 2, so we believe that 2 weeks is sufficient to obtain a sufficient therapeutic effect, but the improvement trend was more pronounced at week 4 than at week 2. We think that the reason for this is that some subjects had a delayed response and were more likely to see improvement at week 4. The severity score of the following seven symptoms was lower at week 2 and week 4 of the treatment period: abdominal pain or abdominal, discomfort, abdominal distension, borborygmi, flatulence, sensation of incomplete defecation, defecation urgency, and straining at stool. In contrast, belching, nausea, and heartburn showed little improvement. A double-blind placebo-controlled trial of ZO-Y60 conducted outside Japan showed that abdominal pain, abdominal distension, stool frequency, borborygmi, and flatulence were improved [8]. The percent disappearance of the respective symptoms after 4-week administration were 55.8%, 51.9%, 59.6%, 61.6%, and 71.1% in the ZO-Y60 arm and 8.2%, 10.2%, 24.4%, 18.4%, and 24.5% in the placebo arm, demonstrating better results for peppermint oil than for placebo. Regarding disappearance of belching, nausea, and heartburn, there was no difference from the placebo arm. Decrease in stool frequency was accompanied by improved stool viscosity (change from watery stool to loose stool or normal stool). When the outcomes of our study in Japanese patients are compared with those of a study conducted outside Japan [8], similar results were obtained in both studies regarding symptoms against which ZO-Y60 was effective and those not effective. The stool frequency score for IBS-C during the treatment period, increased from the screening period, indicating that constipation was improved. The stool form score for IBS-D decreased from the screening period, which indicates that diarrheal stool tended to change to normal stool. We reviewed the major overseas clinical trials of this drug for its effect on IBS-C and IBS-D: Liu et al. study [8] and the Merat et al. study [10]. In the Liu et al. study, it was stated that “Stool consistency improved from watery to soft or normal corresponding to the decrease in stool frequency”. However, it did not clearly state IBS-D or IBS-C, and it was unclear whether the study examined each subtype. In addition, we could not find any description of IBS subtypes in the results of the study by Merat et al. Therefore, although it was possible to compare each symptom with overseas studies, it was difficult to compare by subtype. In addition, the efficacy of this drug is not limited to any subtype in overseas studies, and we think that this drug will be effective for all types of IBS, especially for abdominal pain. In this clinical trial, the rate of appearance of a normal stool increased. Stool form is associated with the bowel transit time [17]. Thus, it is suggested that ZO-Y60 has the effect of normalizing bowel transit time in patients with any subtype of IBS.

Clinical pharmacological studies on the food retention time (gastrointestinal transit time) of peppermint oil have been reported in countries other than Japan [21, 22]. Goerg et al. reported an increase in mouth-to-cecum transit time with peppermint oil in healthy adults [21]. Elongation of whole gut transit time with peppermint oil was also reported in IBS patients [22]. Because only inhibition of rapid whole gut transit time cannot explain the clinical efficacy of peppermint oil on IBS-C patients, an anti-spasmodic effect of l-menthol as the main element of peppermint oil on the smooth muscle cells through the block of Ca^2+^ influx through sarcolemma L-type Ca^2+^ channels [23] is a plausible mechanism of the action in all subtypes of IBS patients. Moreover, transient receptor potential melastatin 8 (TRPM8) receptor is highly expressed in the dendritic cells of the colonic tissue of IBS patients compared with healthy controls and specific stimulation of TRPM8 receptor causes reduction of interleukin (IL)-1β, IL-6, and tumor necrosis factor (TNF)-α release from the biopsy specimens of IBS patients [24]. L-menthol as the main element of peppermint oil is a representative agonist of TRPM8 [24]. Therefore, an anti-spasmodic effect with an anti-inflammatory property is likely to be the origin of the reduction of IBS symptoms by administration of peppermint oil.

In this clinical trial, adverse events occurred in 14 subjects (20.3%). All of these adverse events were mild or moderate in severity, and neither severe nor serious adverse events were observed. Adverse reactions were observed in two subjects (2.9%). One exhibited two events: hypersensitivity and breath odor. The other exhibited eczema. All these events were known, and disappeared during the study period. In a clinical study of ZO-Y60 conducted outside Japan [8], adverse reactions occurred in 3.8% (two of the 52 subjects). One subject experienced heartburn, chewing the capsule allowing it to release the gel in the esophagus. The other exhibited mild skin eruption on both arms. While in our clinical trial hypersensitivity, breath odor, and eczema occurred, these have already been reported with post-marketing safety information on peppermint oil outside Japan. Like the skin eruption observed in the clinical study of ZO-Y60 conducted outside Japan, the eczema and hypersensitivity observed in our clinical trial can be regarded as allergic symptoms induced by ZO-Y60 administration. Skin manifestations are cited as manifestations of hypersensitivity to peppermint oil [25]. Thus, the skin eruption can be considered to be due to hypersensitivity to ZO-Y60. From these findings, we consider that adverse reactions after intake of peppermint oil are not very different between Japanese and other populations.

This study has some limitations. Firstly, a single arm was treated with the active drug. However, together with international data and the present treatment outcomes for just 69 Japanese subjects, ZO-Y60 was approved for the Japanese market in August 2021. Secondly, investigation of each IBS subtype may have been insufficient owing to the small number of patients of each subtype. The results of a placebo-controlled trial of ZO-Y60 conducted outside Japan show its efficacy against abdominal pain, abdominal distension, borborygmi, and flatulence [8]. Our results in Japan also showed efficacy against these common IBS symptoms. It is necessary to pay close attention to post-marketing efficacy and safety information regarding each subtype.

## Conclusion

Peppermint oil ZO-Y60 exerted an overall improvement effect on IBS, regardless of its subtypes, and improved its various symptoms. No severe adverse events occurred. We therefore consider ZO-Y60 a useful therapeutic agent for Japanese patients with IBS.

## Data Availability

Not applicable because this is a clinical study report.

## Abbreviations

95% CI: 95% Confidence interval
IBS: Irritable bowel syndrome
IBS-C: Irritable bowel syndrome with constipation
IBS-D: Irritable bowel syndrome with diarrhea
IBS-M: Irritable bowel syndrome mixed type
OTC: Over-the-counter
SD: Standard deviation
